# The potential of Generative Pre-trained Transformer 4 (GPT-4) to analyse medical notes in three different languages: a retrospective model-evaluation study

**DOI:** 10.1016/S2589-7500(24)00246-2

**Published:** 2025-01

**Authors:** Maria Clara Saad Menezes, Alexander F Hoffmann, Amelia L M Tan, Mariné Nalbandyan, Gilbert S Omenn, Diego R Mazzotti, Alejandro Hernández-Arango, Shyam Visweswaran, Shruthi Venkatesh, Kenneth D Mandl, Florence T Bourgeois, James W K Lee, Andrew Makmur, David A Hanauer, Michael G Semanik, Lauren T Kerivan, Terra Hill, Julian Forero, Carlos Restrepo, Matteo Vigna, Piero Ceriana, Noor Abu-el-rub, Paul Avillach, Riccardo Bellazzi, Thomas Callaci, Alba Gutiérrez-Sacristán, Alberto Malovini, Jomol P Mathew, Michele Morris, Venkatesh L Murthy, Tommaso M Buonocore, Enea Parimbelli, Lav P Patel, Carlos Sáez, Malarkodi Jebathilagam Samayamuthu, Jeffrey A Thompson, Valentina Tibollo, Zongqi Xia, Isaac S Kohane

**Affiliations:** **Department of Biomedical Informatics, Medical School, Harvard University, Boston, MA, USA** (M C S Menezes MD, A F Hoffmann MBA, A L M Tan PhD, P Avillach MD, A Gutiérrez-Sacristán PhD, Prof I S Kohane MD); **Department of Internal Medicine, University of Texas at Southwestern, Dallas, TX, USA** (M C S Menezes); **Office of Informatics and Information Technology, School of Medicine and Public Health, University of Wisconsin-Madison, Madison, WI, USA** (M Nalbandyan MD, M G Semanik MD, T Callaci BSc, J P Mathew PhD); **Computational Medicine and Bioinformatics, Internal Medicine, Human Genetics, Environmental Health** (Prof G S Omenn MD), **Department of Learning Health Sciences** (D A Hanauer MD), **and Department of Internal Medicine and Frankel Cardiovascular Center** (Prof V L Murthy MD), **University of Michigan, Ann Arbor, MI, USA; Division of Medical Informatics and Division of Pulmonary Critical Care and Sleep Medicine, Department of Internal Medicine** (D R Mazzotti PhD), **Department of Surgery** (L T Kerivan MD, T Hill MD), **Research Informatics** (N Abu-el-rub PhD, L P Patel MSc), **and Department of Biostatistics and Data Science** (J A Thompson PhD), **University of Kansas Medical Center, Kansas City, KS, USA; Department of Internal Medicine, University of Antioquia, Hospital Alma Máter de Antioquia, Medellín, Colombia** (A Hernández-Arango MD, J Forero MD, C Restrepo MD); **Department of Biomedical Informatics** (Prof S Visweswaran MD, M Morris BA, M J Samayamuthu MD) **and Department of Neurology** (S Venkatesh BSc, Z Xia MD), **University of Pittsburgh, Pittsburgh, PA, USA; Computational Health Informatics Program** (Prof K D Mandl MD) **and Department of Pediatrics** (F T Bourgeois MD), **Boston Children’s Hospital, Boston, MA, USA; Department of Surgery** (J W K Lee MD) **and Department of Diagnostic Imaging** (A Makmur MBBS), **National University Health System, Singapore; Respiratory Rehabilitation Unit** (M Vigna MD, P Ceriana MD) **and Laboratory of Medical Informatics and Artificial Intelligence** (A Malovini PhD, V Tibollo MEng), **Istituti Clinici Scientifici Maugeri Istituto di Ricovero e Cura a Carattere Scientifico, Pavia, Italy; Department of Electrical, Computer and Biomedical Engineering, University of Pavia, Pavia, Italy** (Prof R Bellazzi PhD, T M Buonocore PhD, E Parimbelli PhD); **Biomedical Data Science Lab, Instituto Universitario de Tecnologías de la Información y Comunicaciones, Universitat Politècnica de València, Valencia, Spain** (C Sáez PhD)

## Abstract

**Background:**

Patient notes contain substantial information but are difficult for computers to analyse due to their unstructured format. Large-language models (LLMs), such as Generative Pre-trained Transformer 4 (GPT-4), have changed our ability to process text, but we do not know how effectively they handle medical notes. We aimed to assess the ability of GPT-4 to answer predefined questions after reading medical notes in three different languages.

**Methods:**

For this retrospective model-evaluation study, we included eight university hospitals from four countries (ie, the USA, Colombia, Singapore, and Italy). Each site submitted seven de-identified medical notes related to seven separate patients to the coordinating centre between June 1, 2023, and Feb 28, 2024. Medical notes were written between Feb 1, 2020, and June 1, 2023. One site provided medical notes in Spanish, one site provided notes in Italian, and the remaining six sites provided notes in English. We included admission notes, progress notes, and consultation notes. No discharge summaries were included in this study. We advised participating sites to choose medical notes that, at time of hospital admission, were for patients who were male or female, aged 18–65 years, had a diagnosis of obesity, had a diagnosis of COVID-19, and had submitted an admission note. Adherence to these criteria was optional and participating sites randomly chose which medical notes to submit. When entering information into GPT-4, we prepended each medical note with an instruction prompt and a list of 14 questions that had been chosen a priori. Each medical note was individually given to GPT-4 in its original language and in separate sessions; the questions were always given in English. At each site, two physicians independently validated responses by GPT-4 and responded to all 14 questions. Each pair of physicians evaluated responses from GPT-4 to the seven medical notes from their own site only. Physicians were not masked to responses from GPT-4 before providing their own answers, but were masked to responses from the other physician.

**Findings:**

We collected 56 medical notes, of which 42 (75%) were in English, seven (13%) were in Italian, and seven (13%) were in Spanish. For each medical note, GPT-4 responded to 14 questions, resulting in 784 responses. In 622 (79%, 95% CI 76–82) of 784 responses, both physicians agreed with GPT-4. In 82 (11%, 8–13) responses, only one physician agreed with GPT-4. In the remaining 80 (10%, 8–13) responses, neither physician agreed with GPT-4. Both physicians agreed with GPT-4 more often for medical notes written in Spanish (86 [88%, 95% CI 79–93] of 98 responses) and Italian (82 [84%, 75–90] of 98 responses) than in English (454 [77%, 74–80] of 588 responses).

**Interpretation:**

The results of our model-evaluation study suggest that GPT-4 is accurate when analysing medical notes in three different languages. In the future, research should explore how LLMs can be integrated into clinical workflows to maximise their use in health care.

**Funding:**

None.

## Introduction

Medical notes are a unique source of information, but transforming narrative text into structured knowledge with traditional natural-language processing generally does not capture the complexity of co-occurring medical problems or disease trajectory over time.^1^ Large-language models (LLMs) can maximise interpretation of clinical information in medical notes. However, use of LLMs to analyse unstructured, free-text medical notes has some challenges, such as accurate extraction of explicit and implicit information. For example, explicit information might include a direct statement (eg, patient reports chest pain for the past 2 h) but implicit information might include a more general statement (eg, patient appeared uncomfortable and was clutching their chest, but denied any discomfort when questioned). The distinction between explicit and implicit information is crucial as LLMs excel at processing explicit details, such as medication information,^2^ but often struggle with the contextual inference necessary for medical decision making.^3^ Variability in documentation style across different health-care providers further contributes to complexities in data processing.^4^

There is encouraging evidence of the potential of LLMs in processing free-text medical notes. For example, a preprint paper showed that Generative Pre-trained Transformer 3 (GPT-3) could decode medical abbreviations and extract detailed medication information from these unstructured documents, such as dose, frequency, duration, and prescription reasons.^2^ A separate preprint paper found that Flan-T5 XXL could identify disease risk factors and symptoms from medical notes alone.^5^ These models could also extract social determinants of health, including data on employment, housing, and social support systems.^6^ However, some tasks are harder for LLMs than others. For example, generating problem lists from progress notes was more difficult for LLMs than summarising radiology reports, patient questions, or doctor–patient dialogues.^7^

All these studies only included medical notes written in English. This limitation is common due to challenges in international medical-note exchange.^8^ However, evaluation of LLMs across multiple countries and languages is essential to assess their effectiveness and adaptability in diverse health-care settings.

We aimed to assess the ability of Generative Pre-trained Transformer 4 (GPT-4) to answer predefined questions after reading medical notes in three different languages. We also aimed to examine the performance of GPT-4 in selecting patients for study enrolment.

## Methods

### Study design

For this retrospective model-evaluation study, we included eight university hospitals from four countries (ie, the USA, Colombia, Singapore, and Italy). The sites were Boston Children’s Hospital, the University of Michigan, the University of Pittsburgh Medical Center, the University of Wisconsin, the University of Kansas Medical Center, Universidad de Antioquia, the National University of Singapore, and Istituti Clinici Scientifici Maugeri. This study was conducted within the 4CE Consortium. Universities already part of the consortium were invited to participate, and we chose the eight sites on the basis of their interest and ability to contribute the necessary data and physician validators. The Department of Biomedical Informatics at Harvard University (Boston, MA, USA) was the coordinating centre.

Each site submitted seven de-identified medical notes related to seven separate patients to the coordinating centre between June 1, 2023, and Feb 28, 2024. Medical notes were written between Feb 1, 2020, and June 1, 2023. Medical notes in Spanish were provided by Universidad de Antioquia, in Italian were provided by Istituti Clinici Scientifici Maugeri, and in English were provided by the six remaining sites.

Each site obtained institutional review board approval from their own institutions and had data-use agreements in place.

### Note selection, de-identification, and submission

We advised participating sites to choose medical notes that met our four inclusion criteria. At time of hospital admission, patients should have been aged 18–65 years, had a diagnosis of obesity, had a diagnosis of COVID-19, and the submitted note should have been an admission note. Adherence to these criteria was optional, and the participating sites randomly chose which medical notes to submit. As a result, we included admission notes, progress notes, and consultation notes. An admission note is the first medical note written when a patient is admitted to a specific medical service (eg, ward or intensive care unit). Progress notes are subsequent medical notes written after the admission note. Consultation notes are written by a specialist who was consulted to assist in the care of a patient, but is not part of the primary medical team responsible for their overall care. A discharge summary is a summary of the entire admission; no discharge summaries were included in this study.

Medical notes were de-identified at participating sites via their preferred method (appendix pp 1–2). All sites were instructed to remove the same protected health information according to the US Health Insurance Portability and Accountability Act guidelines, including those not in the USA.^9^

Patients did not provide written or oral informed consent for their notes to be used. All eight university hospitals involved deemed the study exempt from patient-consent requirements as all notes were de-identified.

### GPT-4 pipeline

We developed a question–answer framework that used the application programming interface (API) of GPT-4 in Python version 3.9.16. Parameters that we explicitly set were a temperature of 0·2, a top-p of 0·95, and a frequency penalty of 0 (appendix p 2). Top-p and frequency penalty are typically not reported, so we used values similar to the default settings of GPT-4’s API. A seed parameter to ensure reproducibility was not available in the API of GPT-4 32k, the model used in this study, and so was not set.

**Figure 1:**
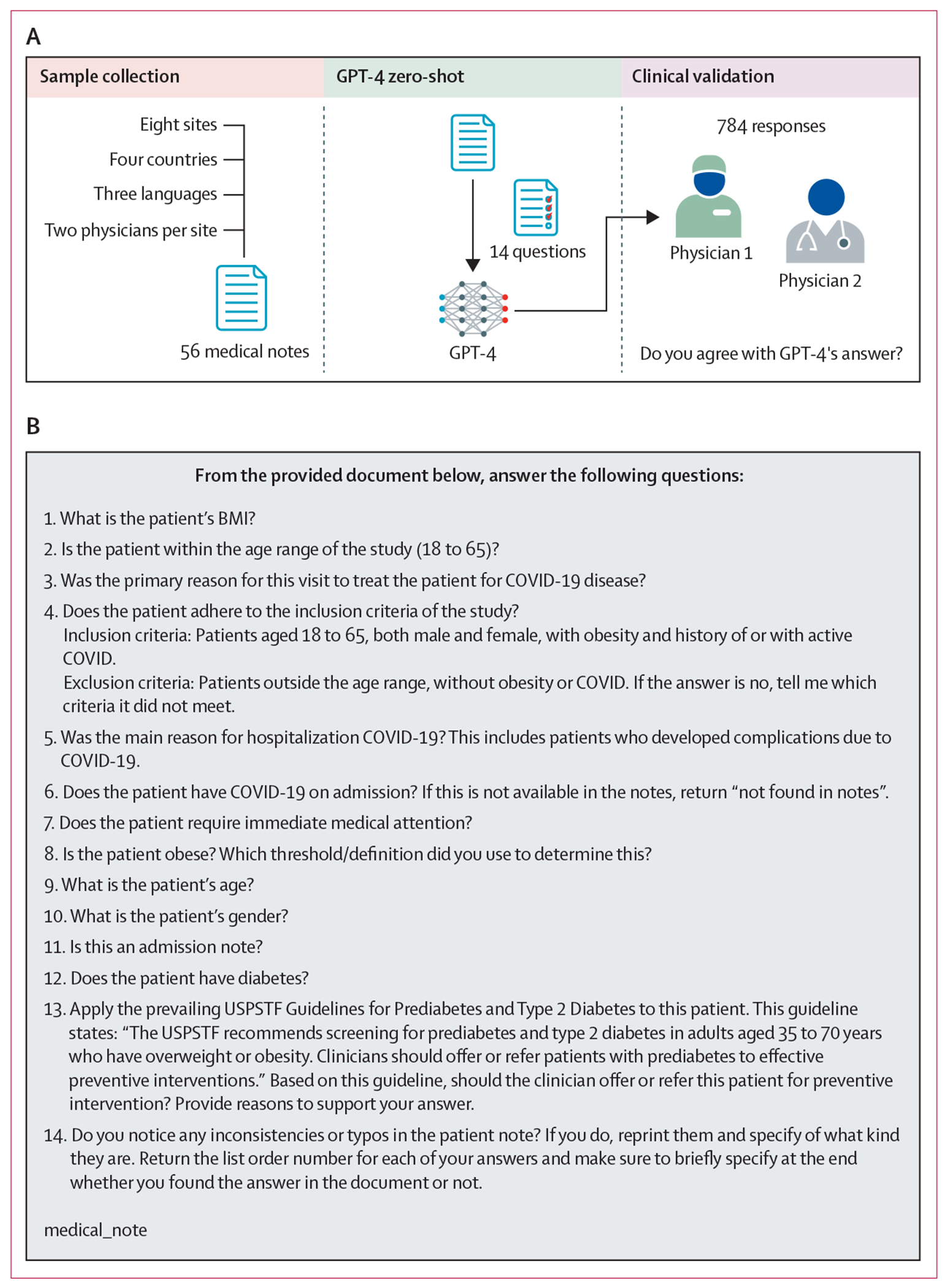
Study design (A) Study processes. (B) Instruction prompts given to GPT-4 with 14 questions chosen by our study group before submission of medical notes. In the prompt, the term medical_note was replaced by the actual medical note. Please note that questions and prompts are verbatim questions and prompts asked to GPT-4, and have not been edited. GPT-4=Generative Pre-trained Transformer 4. USPSTF=US Preventive Services Task Force.

When entering information into GPT-4, we prepended each medical note with an instruction prompt and a list of 14 questions that had been chosen a priori by the study group (GSO, DAH, CS, ZX, and ISK; figure 1). The questions were designed to include key areas of medicalnote information, such as demographics, past medical history, details of current hospitalisation, patient selection for hypothetical study enrolment, application of screening guidelines, and identification of errors in the medical note. We collected gender data by asking the LLM “What is the patient’s gender?”. The LLM was given the medical note for reference and its answer was validated by two physicians.

All 16 physicians, two per site, who clinically validated the responses of GPT-4 participated in creating these questions. A pilot assessment of the questions before the study was not feasible due to our small sample size; it would have required splitting the notes into a subset for the pilot phase. Instead, all 16 physicians evaluated and edited the questions beforehand to ensure clarity and relevance.

Most of the questions were straightforward and specific. The median body of text was approximately 1939 tokens (IQR 1653), depending on the length of the medical note, but was always within the maximum limit of 128 000 tokens set by GPT-4’s API. The texts assembled for each patient note were submitted to the API in the same sequence they were received from participating sites during the study period. Each medical note was individually given to GPT-4 in its original language and in separate sessions, to help reduce the risk of the model retaining or mixing details from one session to another; the questions were always given in English. GPT-4 translated medical notes written in Spanish and Italian and provided answers in English. No additional modifications to the prompts in non-English texts were necessary.

To avoid selection bias, we aimed to recruit a broad range of universities by announcing our aims and study to the 4CE Consortium, which includes 40 academic hospitals across four continents. To minimise framing bias, all question prompts were made available for review by all authors.

### Clinical validation

At each site, two physicians independently validated responses by GPT-4 and responded to all 14 questions in a free-text format, followed by a Yes or No answer to the question “Do you agree with GPT-4’s answer?”. Each pair of physicians evaluated responses from GPT-4 to the seven medical notes from their own site only. Physicians were not masked to responses from GPT-4 before providing their own answers but were masked to responses from the other physician, to ensure independence (appendix p 2). All physicians self-reported being proficient in English.

### Statistical analysis

The decision to request seven notes was made heuristically to ensure a manageable and consistent sampling approach across institutions. Regarding the number of sites and languages, we used the maximum available.

To explore factors contributing to different physician opinions regarding responses from GPT-4, we examined responses for which one physician agreed with GPT-4 but the other did not. These situations were categorised into three groups. We identified an extraction problem if the disagreement happened because one physician overlooked explicit information in the medical note that the other physician noted, such as a patient’s previous diagnosis. We identified an inference problem if the difference in physicians’ perception of a response from GPT-4 was due to one physician making different inferences from the medical note than the other physician, such as different opinions regarding the need for urgent medical care based on symptoms. If both physicians provided the same free-text answer to the question but differed in their agreement with the response from GPT-4 due to unknown reasons, we categorised the situation as different levels of agreement with GPT-4.

To decipher responses in which GPT-4 most likely gave an erroneous response, we explored scenarios in which both physicians disagreed with its response. We identified an extraction problem if GPT-4 overlooked explicit information that was present in the medical note. We identified an inference problem when GPT-4 made or failed to make logical inferences that differed from those made by both physicians. We identified a hallucination if GPT-4 generated information that was not explicitly present or could not be reasonably inferred from the note.

To assess the ability of GPT-4 to select patients for hypothetical study enrolment, we evaluated its sensitivity and specificity in identifying medical notes that met our inclusion criteria. For this analysis, only responses for which physicians unanimously agreed or disagreed with GPT-4 were considered, to compare against GPT-4’s performance. We did not use question 4 (figure 1B) in this evaluation because we observed responses in which GPT-4 provided the correct answer for incorrect reasons. For example, GPT-4 might have stated that the patient did not meet the eligibility criteria of the study because they did not have a diagnosis of obesity when the actual reason was that the patient was not in the specified age range.

We noted a posteriori that GPT-4’s accuracy in identifying admission notes was lower than in the other three criteria (ie, age, obesity, and COVID-19). Consequently, we decided to conduct a subgroup analysis excluding the admission note criterion, as this criterion related to the structure of the note rather than its content. There were no missing data in this study.

There was a miscommunication between the coordinating centre and the National University of Singapore, resulting in an unmasked validation of responses by physicians, who compared their responses before submission. To account for this unmasked validation, we conducted a sensitivity analysis excluding the National University of Singapore from the pooled assessment.

We used χ^2^ to assess agreement between physicians and GPT-4 across sites, languages, and questions. All analyses were done in RStudio version 2022.7.1.554.

### Role of the funding source

There was no funding source for this study.

## Results

We collected 56 medical notes from eight sites in four countries (ie, the USA, Colombia, Singapore, and Italy). Of these, 42 (75%) notes were in English, seven (13%) were in Italian, and seven (13%) were in Spanish (appendix p 3). For each medical note, GPT-4 responded to 14 questions, resulting in 784 responses.

**Figure 2:**
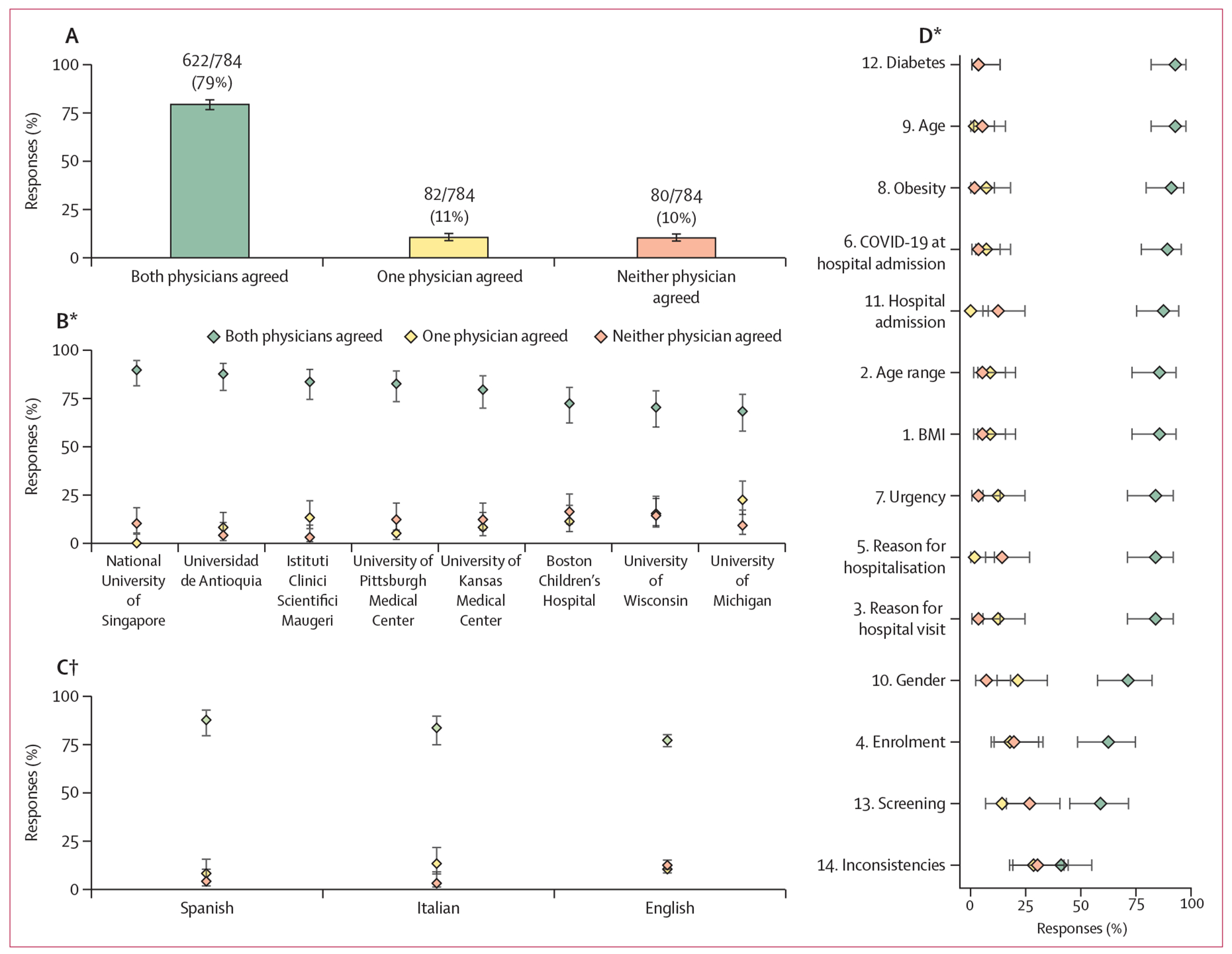
Clinical validation of responses from GPT-4 (A) Agreement of two separate physicians with GPT-4. (B) Agreement with responses by site. (C) Agreement with responses by language. (D) Agreement with results by question. The full list of questions is provided in figure 1. Both agreed refers to when both physicians answered Yes to the question “Do you agree with GPT-4’s answer?” after reading its response. One agreed refers to when one physician agreed with the response from GPT-4, but the other did not. Neither agreed refers to when both physicians did not agree with the response from GPT-4. GPT-4=Generative Pre-trained Transformer 4. *p<0·0001. †p=0·0073.

In 622 (79%, 95% CI 76–82) of 784 responses, both physicians agreed with GPT-4. In 82 (11%, 8–13) responses, only one physician agreed with GPT-4. In the remaining 80 (10%, 8–13) responses, neither physician agreed with GPT-4 (figure 2A; appendix pp 3–4). When excluding the National University of Singapore, both physicians agreed with GPT-4 in 534 (78%, 75–81) of 686 responses, only one physician agreed with GPT-4 in 82 (12%, 10–15) responses, and neither physician agreed with GPT-4 in 70 (10%, 8–13) responses.

Regarding language, both physicians agreed with GPT-4 more often for medical notes written in Spanish (86 [88%, 95% CI 79–93] of 98 responses) and Italian (82 [84%, 75–90] of 98 responses) than in English (454 [77%, 74–80] of 588 responses; figure 2C). Although notes written in Spanish and Italian were shorter than those written in English, length of notes did not affect physicians’ agreement with GPT-4 (appendix pp 4–5). All notes written in Spanish and Italian were admission notes; however, type of note did not affect the likelihood of physicians agreeing with GPT-4 (appendix p 6). Analyses are available by question (figure 2D) and by country (appendix p 7).

**Figure 3:**
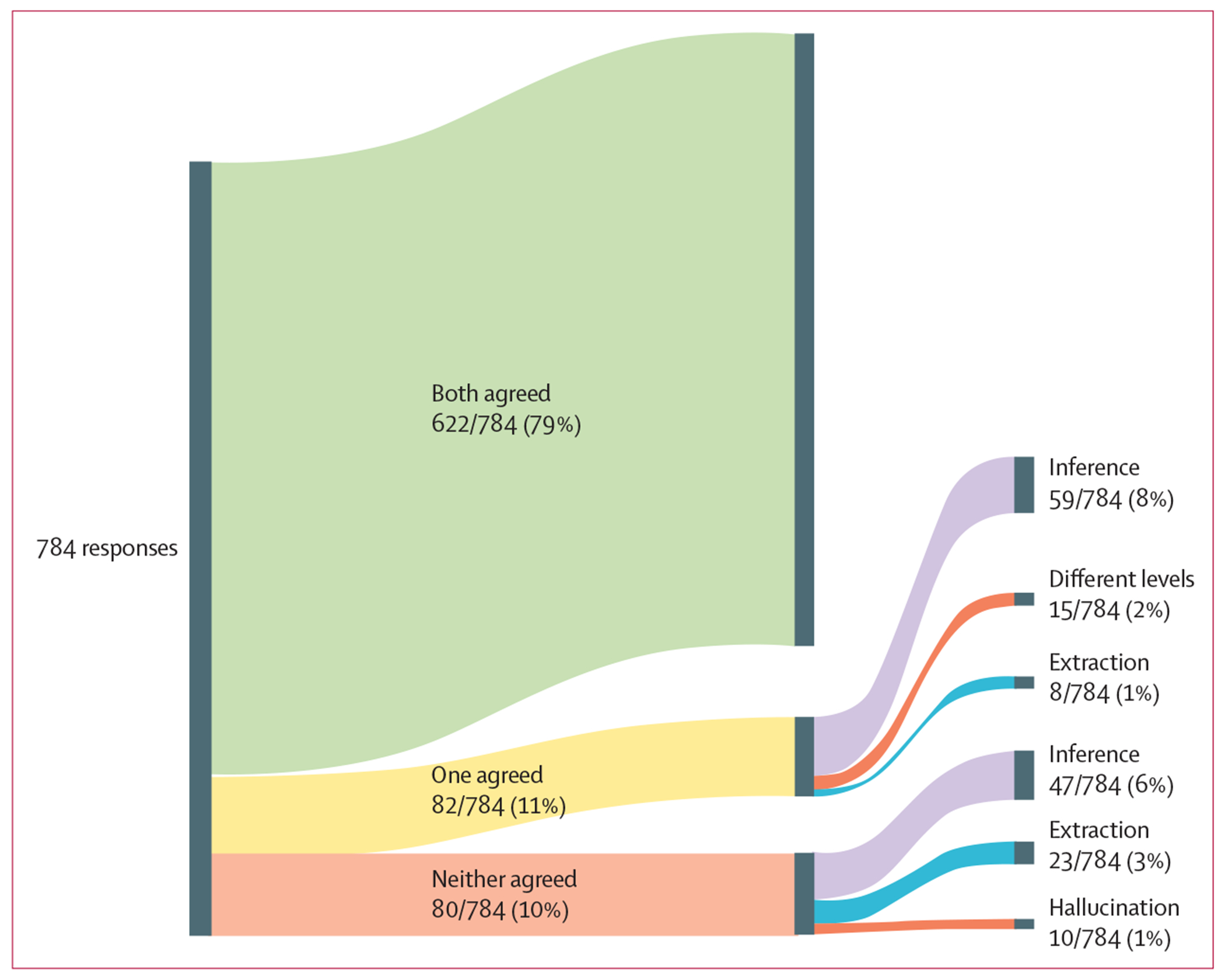
Agreement between physicians and GPT-4 Nodes do not sum to 100% due to rounding. Both agreed refers to when both physicians answered Yes to the question “Do you agree with GPT-4’s answer?” after reading its response. One agreed refers to when one physician agreed with the response from GPT-4, but the other did not. Neither agreed refers to when both physicians did not agree with the response from GPT-4. GPT-4=Generative Pre-trained Transformer 4.

We analysed the 82 responses for which only one physician agreed with GPT-4 (table 1; figure 3). In 59 (72%) of these responses, disagreement was due to an inference problem. For example, upon seeing a mention of recent COVID-19 infection in a medical note, one physician deduced that the patient did not currently have COVID-19. By contrast, the other physician and GPT-4 considered the current COVID-19 status of the patient to be unknown. In 15 (18%) of 82 responses, physicians had different levels of agreement with GPT-4. For example, for one medical note, both physicians responded that hospital admission of the patient was due to COVID-19 causing hypoxaemia, whereas GPT-4 specified that admission was for hypoxaemia with a positive COVID-19 test. Because there was no explicit connection between COVID-19 and hypoxaemia in the response from GPT-4, one physician considered its assessment to be sufficient, whereas the other physician found it inadequate. In eight (10%) of 82 responses, disagreement was due to an extraction problem. For example, in one response, a physician overlooked the documented history of COVID-19 of a patient. By contrast, the other physician noticed this history (appendix p 8).

In the 59 responses we categorised as an inference problem, we identified some common inference-based disagreements between physicians. 12 (20%) of the 59 disagreements were due to varying interpretations of gender; some physicians differentiated between gender and sex and others did not. By contrast, GPT-4 consistently equated gender and sex. 11 (19%) disagreements were due to different assumptions regarding the ages of de-identified patients, inferred by physicians from indirect clues such as admission to an adult service or pregnancy status. Of the most common disagreements, six (10%) were due to diagnosis of COVID-19 or its complications, five (9%) were due to need for urgent medical attention, and five (9%) were due to interpretation of diabetes screening guidelines.

We analysed the 80 responses for which both physicians disagreed with GPT-4 (table 2; figure 3). In 47 (59%) of these responses, disagreement was due to an inference problem. For example, both physicians deduced that a patient admitted with multisystemic inflammatory syndrome and a positive COVID-19 test was more likely to have been admitted to hospital for a COVID-19 complication because of the potential causality between the two, a connection that GPT-4 did not make. In 23 (29%) responses, disagreement was due to an extraction problem. For example, GPT-4 did not identify a patient’s age that was explicitly stated as 30 years at the beginning of the medical note, a detail that the physicians successfully extracted. In ten (13%) responses, disagreement was due to a hallucination problem. For example, when answering the question regarding whether the patient had COVID-19 on hospital admission, GPT-4 hallucinated and answered that the patient had COVID-19, whereas both physicians concurred that COVID-19 was not mentioned in the note. The remaining nine responses that we categorised as a hallucination problem were related to age. GPT-4 accurately established the ages of patients. However, it made illogical statements regarding the age range, such as stating that “the patient is 63 years old, which is outside the age range of 18 to 65” (appendix p 9).

In the 47 responses we categorised as an inference problem, we identified some common inference-based disagreements between GPT-4 and both physicians. For 19 (40%) of these responses, GPT-4 struggled with inferring information from the template structure, such as recognising a medical note as an admission note on the basis of its format or identifying placeholders as de-identified data. For five (11%) responses, GPT-4 could not infer that people with diabetes did not need to be screened for this disease. In another five (11%) responses, GPT-4 failed to recognise complications from COVID-19. In a further five (11%) responses, GPT-4 was unable to establish the primary reason for the hospital visit or the urgency of medical attention.

**Figure 4:**
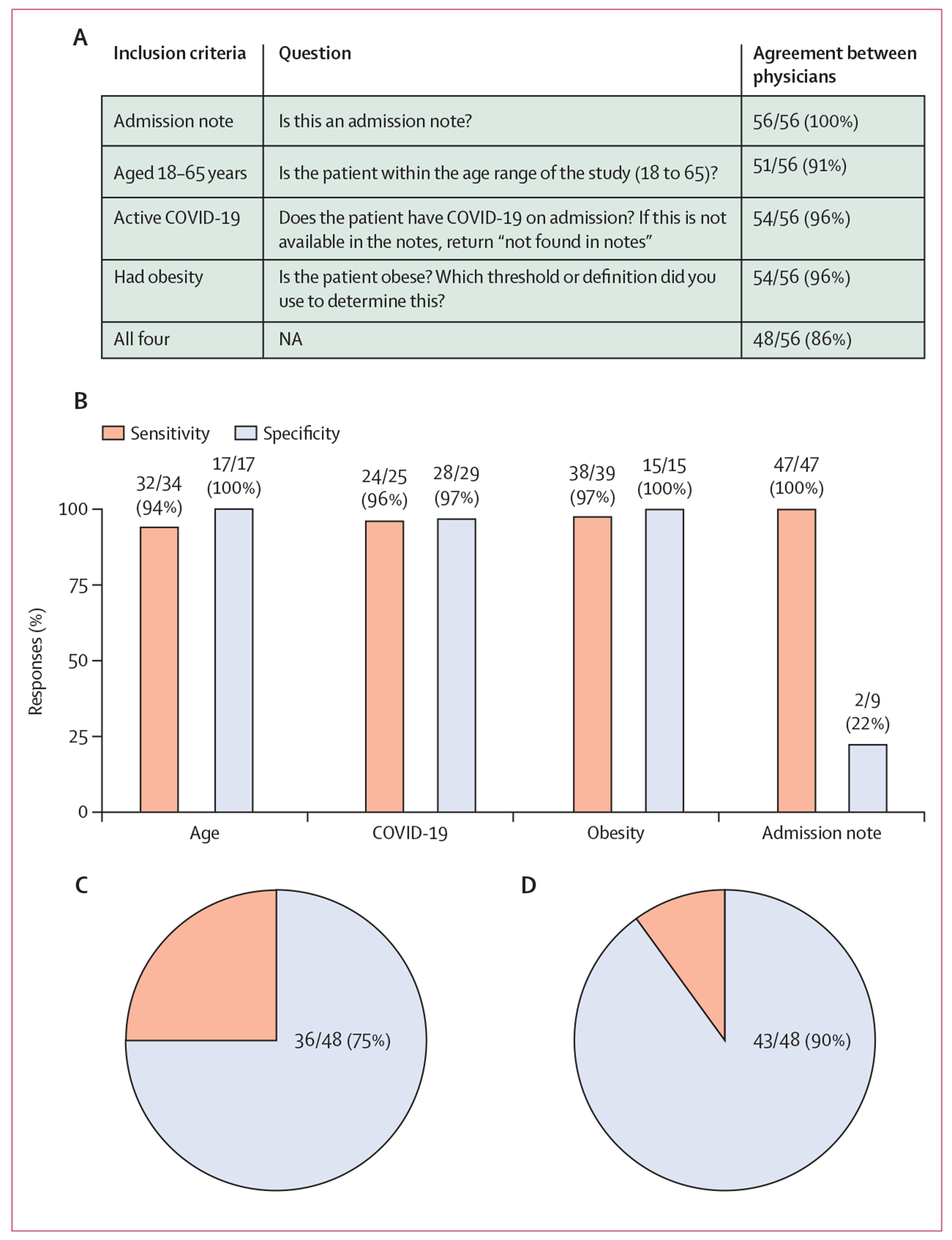
Ability of GPT-4 to select patients for hypothetical study enrolment (A) Inclusion criteria. We only included questions for which both physicians agreed or disagreed with GPT-4 in this analysis. Please note that questions are verbatim questions asked to GPT-4 and to physicians, and have not been edited. (B) Sensitivity and specificity, by inclusion criteria. Denominators for each criterion are numerators for that criterion in the column showing agreement between physicians in panel A. For example, we considered 51 notes in the analysis for age, 34 of which were within the age range of the study and were used to calculate sensitivity and 17 of which were not within the age range of the study and were used to calculate specificity. (C) Proportion of times GPT-4 was able to correctly provide all four inclusion criteria in a single patient. (D) Proportion of times GPT-4 was able to correctly provide all three inclusion criteria (excluding admission note) in a single patient. GPT-4=Generative Pre-trained Transformer 4. NA=not applicable.

Of the 56 medical notes, physicians unanimously agreed that seven (13%) met inclusion criteria for hypothetical study enrolment and 41 (73%) did not meet inclusion criteria. For eight (14%) notes there was disagreement between physicians about whether they met inclusion criteria. The sensitivity of GPT-4 was 100% for admission notes, 97% for obesity, 96% for COVID-19, and 94% for age (figure 4). The specificity of GPT-4 was 100% for age, 100% for obesity, and 97% for COVID-19. However, the specificity for admission notes was substantially lower at 22% (figure 4).

When considering all four inclusion criteria, GPT-4 accurately identified all four in 36 (75%, 95% CI 62–88) of 48 responses, but was correct in only three of the criteria in the remaining 12 (15%, 13–37) responses (figure 4C). When we excluded the admission-note criteria, GPT-4 accurately identified all three criteria in 43 (90%, 81–98) of 48 responses, but was correct in only two of the criteria in the remaining five (10%, 2–19) responses (figure 4D).

## Discussion

The results of our model-evaluation study suggest that GPT-4 is accurate when analysing medical notes in three different languages, even without prompt engineering.

Although the data used to train GPT-4 have not been made public, we assume that they consist predominantly of English text on the basis of a preprint paper.^10^ Thus, our finding that GPT-4 analysed medical notes in Italian and Spanish better than notes in English was surprising. This finding could be because notes in the USA tend to be more complex and longer.^11^ However, we could not identify a correlation between note length and performance of GPT-4.

GPT-4 had minimal issues extracting explicit information. The most common reason why physicians disagreed with responses from GPT-4 was its inability to infer implicit information. GPT-4 is a general-purpose model; a preprint paper has suggested that LLMs specifically optimised for medical tasks are likely to overcome this limitation.^12^ The challenge in inferring information was possibly exacerbated by our use of a low temperature; however, this temperature probably helped to minimise hallucinations. There is no established guidance on the optimal temperature for use of GPT-4 in clinical analysis, and previous assessments, including a preprint paper, have used different strategies.^13–15^

When selecting patients for hypothetical study enrolment, GPT-4 was strong in criteria reliant on explicit information, such as age, presence of an obesity diagnosis, and COVID-19 at hospital admission. Our results align with 2024 data suggesting that GPT-4 can reliably extract clinical inclusion and exclusion criteria from medical notes, with an accuracy of 98–100%.^16^ This accuracy is similar to traditional methods for patient selection for study enrolment that rely on study staff reviewing medical charts, shown to have an accuracy of 91–100% in a preprint paper.^16^

In our study, GPT-4 struggled to identify whether a note was an admission note, potentially because that would require an understanding of implicit details such as document structure. However, information such as note type is often readily available as metadata, so could easily be provided to LLMs if needed. Furthermore, the substantial difference between sensitivity and specificity regarding this criterion suggests a framing bias in which GPT-4 tended to affirmatively answer the question “Is this an admission note?”, similar to varying responses from GPT-3 regarding question framing shown in a preprint paper.^17^

The main limitation of our study was its small sample size. Gathering medical notes from different institutions was challenging as most institutions worldwide do not allow their notes to be input into LLMs, as shown in previous publications that include preprint papers.^2.5–7^ However, we were able to reach an agreement with each site to share only seven notes as a starting point for future work. Moreover, agreement between physicians and GPT-4 was consistent across the five US sites, indicating that each site could represent the performance of GPT-4 across the USA. We hope that the notes from Singapore, Spain, and Italy could be indicative of how this LLM would perform in the respective countries, but larger studies are needed to substantiate this notion. We hope our results will encourage further, larger studies; opportunities for insight and collaboration will be clear as a result, as opposed to focusing only on the considerable administrative and regulatory effort.

Our study had further limitations. First, submitted medical notes were chosen randomly by participating sites, leading to concerns about selection bias. Second, we only considered one LLM. Although GPT-4 is strong in summarising information, other models could provide different results.^7^ Third, GPT-4 is not an open-source model, so scaling up a study like ours would be logistically challenging, for example in having our clinicians fully validate outcomes, and would raise concerns regarding data privacy and cost. Fourth, we were limited by the 14 questions we chose to evaluate GPT-4; the answers to these questions might not accurately reflect its ability to analyse medical notes on a broad scale. Fifth, allowing physicians to see responses from GPT-4 before providing their own could affect the objectivity and independence of their assessments, as they could have been guided by its answers. Sixth, the way we asked the question “Do you agree with GPT-4’s answer?” could have introduced confirmation bias; future studies should ask questions in a neutral way. Finally, there was a miscommunication between the coordinating centre and the National University of Singapore, leading to unmasked validation of GPT-4’s responses by physicians, which could have introduced bias.

Our model-evaluation study is the first to use an LLM to analyse medical notes in three different languages, extracting explicit information and interpreting what is implied through context.^18^ Our findings highlight the potential of LLMs, such as GPT-4, in analyses of medical notes. We found that GPT-4 struggled the most with making connections using implicit information in medical notes, emphasising the need for further research to optimise LLMs for this type of task. In the future, research should explore how LLMs can be integrated into clinical workflows to maximise their use in health care.

## Supplementary Material

Supplementary Material

## Figures and Tables

**Table 1:** Clinical validation when only one physician agreed with GPT-4

	Number of responses	Question	GPT-4	Physician 1	Physician 2
Different levels	15/82 (18%)	Was the main reason for hospitalization COVID-19? This includes patients who developed complications due to COVID-19	Main reason for hospitalisation was hypoxia, but the patient had COVID-19 disease	Primary reason for admission was COVID-19, resulting in hypoxic respiratory failure*	Hypoxaemia due to recurrent COVID-19 infection†
Extraction	8/82 (10%)	Does the patient adhere to the inclusion criteria of the study? Patients aged 18 to 65, both male and female, with obesity and history of or with active COVID-19	No, the patient did not adhere to inclusion criteria; the patient did not have an active COVID-19 infection (found in medical notes)	Patient met the definition as they had a history of recent COVID-19 infection*	Female patient with obesity aged 19 years†‡
Inference	59/82 (72%)	Does the patient have COVID-19 on admission?	Not found in medical notes	No, the phrase recent COVID-19 infection implied no current COVID-19 infection*	Same answer as GPT-4†

Please note that questions are verbatim questions asked to GPT-4 and to physicians, and have not been edited. GPT-4=Generative Pre-trained Transformer 4.

* No agreement with GPT-4.

† Agreement with GPT-4.

‡ Physician 2 overlooked that this patient had had COVID-19 previously and so met inclusion criteria.

**Table 2:** Clinical validation when neither physician agreed with GPT-4

	Number of responses	Question	GPT-4	Physician 1	Physician 2
Hallucination	10/80 (13%)	Does the patient have COVID-19 on admission? If this is not available in the notes, return “not found in notes”	Patient had COVID-19 on hospital admission, as mentioned in the medical notes	There was no mention of COVID-19 in the medical notes	COVID-19 was not explicitly mentioned
Extraction	23/80 (29%)	Is the patient within the age range of the study (18 to 65)?	Patient’s age was not provided in the medical notes, so whether they were within the age range was unclear	30 years	Yes, 30 years
Inference	47/80 (59%)	Was the main reason for hospitalization COVID-19? This includes patients who developed complications due to COVID-19	No, the main reason for hospitalisation was not COVID-19, but multisystemic inflammatory syndrome (found in medical notes)	The main reason for hospitalisation was complications related to COVID-19 (ie, multisystemic inflammatory syndrome)	Multisystemic inflammatory syndrome is a complication of COVID-19

Please note that questions are verbatim questions asked to GPT-4 and to physicians, and have not been edited.

GPT-4=Generative Pre-trained Transformer 4.

## Data Availability

The prompt used to query GPT-4 is available in the manuscript. The code can be found at https://github.com/a-hoffmann/4ce_llm/blob/main/4CE_pipeline_final.ipynb. Medical notes and the full list of responses from GPT-4 will not be shared to protect the privacy of the patients. During the preparation of this work, the authors used GPT-4 to assist in writing the manuscript. After using GPT-4, the authors reviewed and edited the content as needed and take full responsibility for the content of the publication.

## Research in context

### Evidence before this study

We searched PubMed from database inception to April 29, 2024, without language restrictions to find studies using Generative Pre-trained Transformer 4 (GPT-4) to analyse medical notes. We used the search terms “GPT-4” AND “medical note” OR “records” and reviewed 26 studies. All of these studies showed the potential of GPT-4 for various medical tasks, such as board-examination performance. However, only nine used GPT-4 to analyse medical notes. In these studies, GPT-4 was proficient in extracting medication information, assessing social determinants of health, and summarising medical reports.

### Added value of this study

To the best of our knowledge, no previous study has explored the ability of GPT-4 to analyse medical notes in different languages. We collected medical notes from eight different health institutions in four countries, written in English, Spanish, or Italian. We asked GPT-4 to answer predefined questions about these notes and its responses were clinically validated by two independent physicians at each site. Our findings indicate that GPT-4 accurately analysed medical notes in all three tested languages. Most failures of GPT-4 occurred when the task involved understanding implicit information within medical notes, such as recognising that one disease is a complication of another. Our results also show that GPT-4 can reliably select patients for study enrolment.

### Implications of all the available evidence

Our findings emphasise the potential of GPT-4 to automate the analysis of medical notes in multiple languages. GPT-4 performed well at selecting patients for studies, but might be less suited for more complex tasks that require a nuanced medical understanding. Further research is needed to enhance GPT-4’s performance in more complex medical tasks (eg, requiring deep clinical reasoning, contextual understanding, or multifaceted decision making), including establishing the optimal model temperature for tasks requiring greater inference, deciding between general or fine-tuned large-language models for nuanced medical tasks, and identifying the most effective ways to prompt these models.
