# Supplementary material for: The potential of Generative Pre-trained Transformer 4 (GPT-4) to analyse medical notes in three different languages: a retrospective model-evaluation study
Source: Lancet Digit Health. Author manuscript; Available in PMC 2025 Jun 22. (PMC12182955; doi:10.1016/S2589-7500(24)00246-2)
Supplement: Supplementary Material [file NIHMS2087720-supplement-Supplementary_Material.pdf]

THE LANCET
Digital Health

Supplementary appendix

This appendix formed part of the original submission and has been peer reviewed. We post it as supplied by the authors.

Supplement to: Menezes MCS, Hoffmann AF, Tan ALM, et al. The potential of Generative Pre-trained Transformer 4 (GPT-4) to analyse medical notes in three different languages: a retrospective model-evaluation study. *Lancet Digit Health* 2025; **7:** e35–43.

APPENDIX

Table of Contents

APPENDIX 1

Supplementary Table 1. De-identification Techniques by Site. *1*

Supplementary Table 2. Evidence on GPT-4 Parameter Choices for Medical Note Reading Studies. *2*

Supplementary Table 3. Specialization and Level of Experience of Physicians. *2*

Supplementary Table 4. General Characteristics of Medical Notes, Stratified by Site. *3*

Supplementary Table 5. Agreement Between GPT-4 and Physicians, Stratified by Site. *3*

Supplementary Figure 1. Agreement Between at Least One Physician and GPT-4, Stratified by Site. *4*

Supplementary Table 6. General Characteristics of Medical Notes, Stratified by Language. *4*

Supplementary Figure 2. Influence of Note Length on Physician Agreement with GPT-4's Responses, Stratified by Language. *5*

Supplementary Figure 3. Influence of Note Type on Physician Agreement with GPT-4's Responses. *6*

Supplementary Figure 4. Influence of Country on Physician Agreement with GPT-4's Responses. *7*

Supplementary Figure 5. Analysis of Discrepancies: When Only One Physician Agreed with GPT-4 by Language, Site, and Question. *8*

Supplementary Figure 6. Analysis of Discrepancies: When Neither Physician Agreed with GPT-4 by Language, Site, and Question. *9*

Supplementary Table 7. Members of the 4CE Consortium. *9*

Supplementary Table 1. De-identification Techniques by Site.

Site	Names	Dates	Age
BCH	De-identified as yyy	De-identified as DATET1	Present, not de-identified
ICSM	De-identified as DR001 (Doctor's name) and patient's name not mentioned in the notes*	Present, not de-identified	Not present in the notes
KUMC	De-identified as [PERSON]	Present, not de-identified	Present, not de-identified
NUS	Doctor's name present and patient's name not mentioned in the notes*	De-identified as a blank space	Present, not de-identified
UDEA	De-identified as a blank space	Present, not de-identified	Present, not de-identified
UMICH	De-identified as [NAME]	De-identified as [DATE]	De-identified as [AGE]

UPMC	De-identified as [PERSONALNAME]	De-identified as a blank space	Present, not de-identified
WISC	De-identified as Name	De-identified to only include the year	Present, not de-identified

*Patients were consistently referred to as patients rather than by their names.

Supplementary Table 2. Evidence on GPT-4 Parameter Choices for Medical Note Reading Studies.

Paper	Temperature	Top-p	Frequency Penalty
Agrawal 2023[3]	0	Not reported	Not reported
Ahsan 2023[4]	Not reported	Not reported	Not reported
Guevara 2024[5]	0	Not reported	Not reported
Van Veen 2024[6]	0.1*	Not reported	Not reported
Unlu 2024[15]	0	Not reported	Not reported

*Sensitivity analysis was performed and 0.1 was superior to 0.5 and 0.9.

Supplementary Table 3. Specialization and Level of Experience of Physicians.

Validators	Site	Specialization	Experience
Florence T Bourgeois MD MPH	BCH	Pediatrics, Emergency Medicine	> 5 years
Kenneth D Mandl MD MPH	BCH	Pediatrics	> 5 years
Matteo Vigna MD	ICSM	Pulmonology	> 5 years
Piero Ceriana MD	ICSM	Pulmonology	> 5 years
Lauren T Kerivan MD*	KUMC	Surgery	< 5 years
Terra Hill MD*	KUMC	Surgery	< 5 years
James WK Lee MD MMED	NUS	Surgery	> 5 years
Andrew Makmur MBBS MMed	NUS	Radiology	> 5 years
Carlos Andrés CR Restrepo Castro MD	UDEA	Internist	> 5 years
Julian Forero MD	UDEA	Internist	> 5 years
Gilbert S Omenn MD MPH	UMICH	Internist	> 5 years
David A Hanauer MD MS	UMICH	Pediatrics	> 5 years
Shyam Visweswaran MD PhD	UPMC	Neurology	> 5 years
Shruthi Venkatesh BS**	UPMC	None	< 5 years
Michael G Semanik MD MS	WISC	Pediatric Nephrologist	> 5 years

Marine Nalbandyan MD PhD	WISC	Data Scientist	> 5 years

As shown above, most physician validators have more than five years of experience. The exceptions are two surgery resident physicians at KUMC* and one MD-PhD candidate at UPMC**. More than half of the physician validators have more than one degree.

Supplementary Table 4. General Characteristics of Medical Notes, Stratified by Site.

Site	Sample Size (N)	Admission Notes (N)*	Token Length (median, IQR)
BCH	7	1	1933 (750)
NUS	7	4	942 (450)
UMICH	7	7	4358 (2495)
UPMC	7	7	2642 (656)
WISC	7	7	2609 (943)
KUMC	7	7	2650 (1225)
UDEA	7	7	928 (290)
ICSM	7	7	1068 (636)
Overall	56	47	1939 (1653)

*Though the length of the notes varied significantly across sites, their structure was somewhat consistent. Most notes included in this study were admission notes (47/56). The admission notes from the US (BCH, KUMC, UMICH, UPMC, WISC), Colombia (UDEA), and Singapore (NUS) had very similar structures: chief complaint, history of present illness, objective, assessment, and plan. The admission notes from Italy had a slightly different structure: past medical history, recent medical events, diagnosis, and therapy. Other submitted notes included three progress notes from NUS, five progress notes from BCH, and one consult note from BCH, all following the subjective, objective, assessment, and plan format.

Supplementary Table 5. Agreement Between GPT-4 and Physicians, Stratified by Site.

Site	Both Agreed (%)	One Agreed (%)	At Least One Agreed (%)
BCH	72% (95%CI 62-81%)	11% (95%CI 6-20%)	84% (95%CI 76-91%)
NUS	90% (95%CI 82-95%)	-	90% (95%CI 82-95%)
UMICH	68% (95%CI 58-77%)	22% (95%CI 15-32%)	91% (95%CI 85-97%)
UPMC	83% (95%CI 73-89%)	5% (95%CI 2-12%)	88% (95%CI 81-94%)
WISC	70% (95%CI 60-79%)	15% (95%CI 9-24%)	86% (95%CI 79-92%)
KUMC	80% (95%CI 70-87%)	8% (95%CI 4-16%)	88% (95%CI 81-94%)
UDEA	88% (95%CI 79-93%)	8% (95%CI 4-16%)	95% (95%CI 92-100%)

ICSM	84% (95%CI 75-90%)	13% (95%CI 8-22%)	96% (95%CI 93-100%)
Overall	79% (95%CI 76-82%)	10% (95%CI 8-13%)	90% (95%CI 88-92%)

Supplementary Figure 1. Agreement Between at Least One Physician and GPT-4, Stratified by Site.

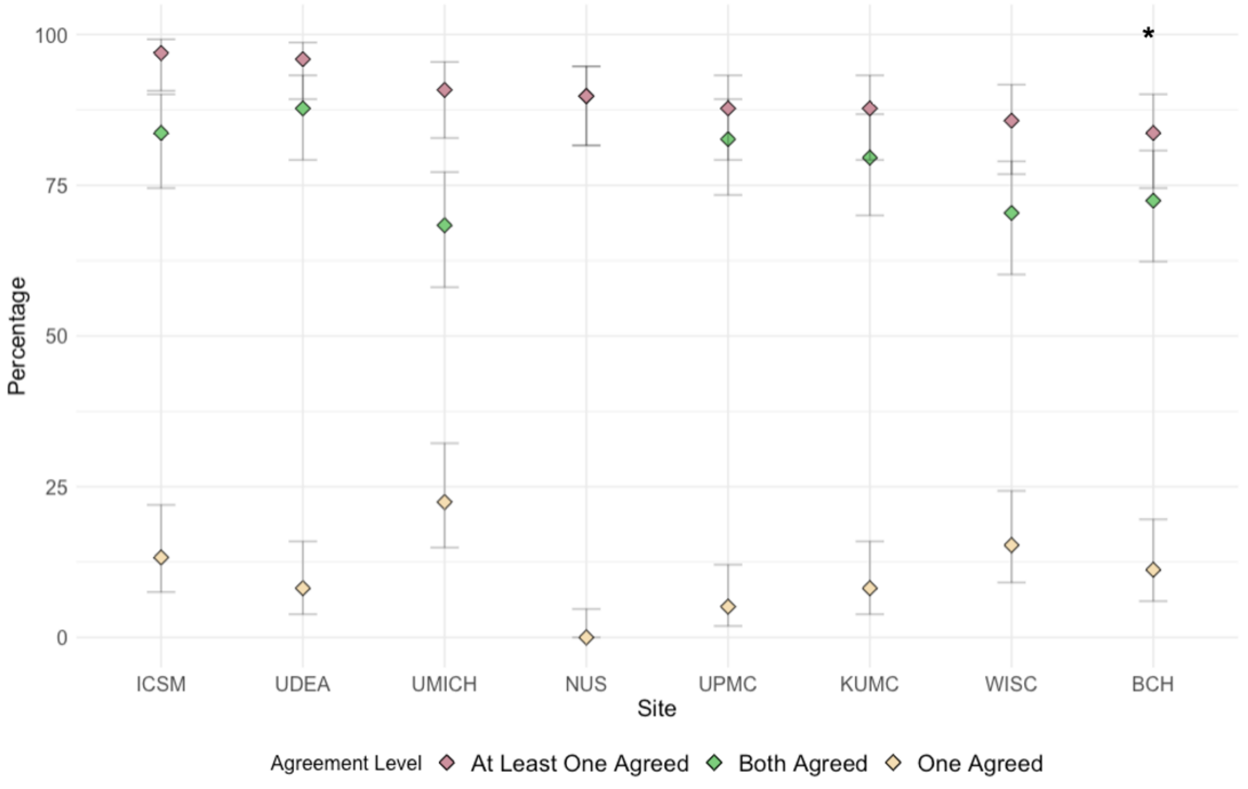

Legend: The sites are arranged in descending order based on the frequency of agreement of at least one physician with GPT-4's responses. *P value 0·02 (Chi-squared test).

Supplementary Table 6. General Characteristics of Medical Notes, Stratified by Language.

Language	Sample Size (N)	Admission Notes (N)	Token Length (median, IQR)
English	42	33	2393 (1421)
Italian	7	7	1068 (636)
Spanish	7	7	928 (290)
Overall	56	47	1939 (1653)

Supplementary Figure 2. Influence of Note Length on Physician Agreement with GPT-4's Responses, Stratified by Language.

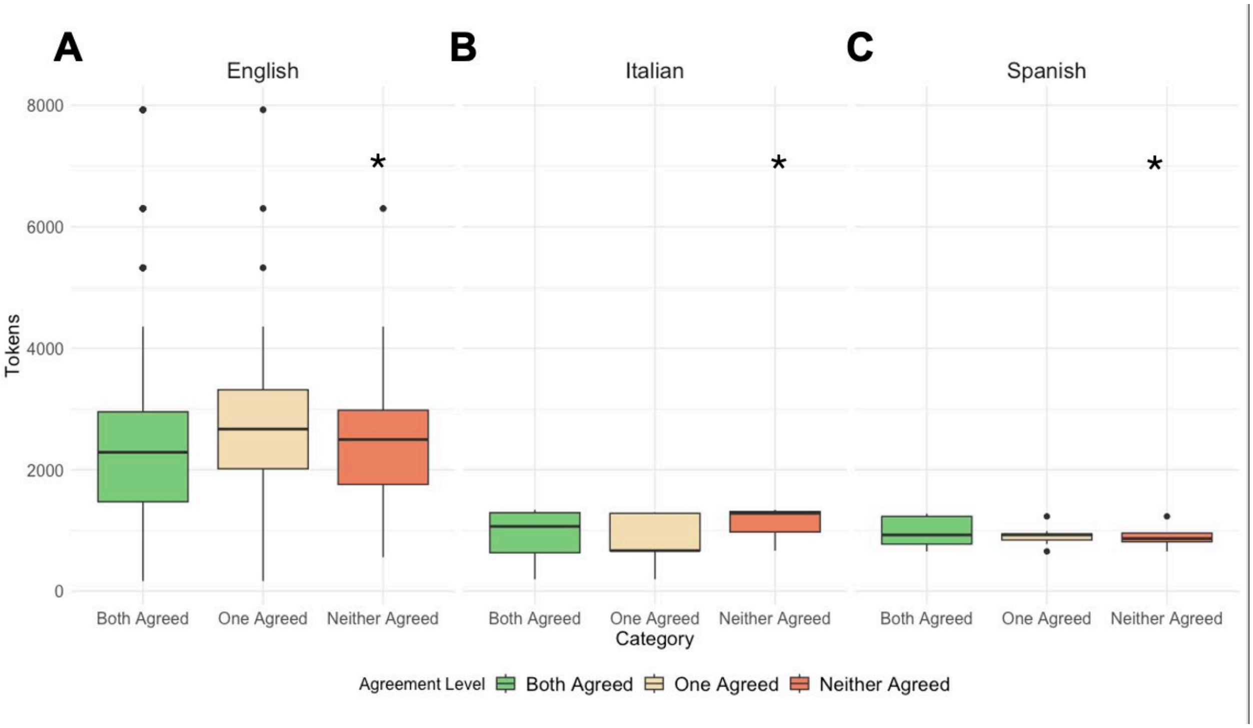

Legend: From left to write: (A) English. Median token lengths and IQRs are as follows: Both Agreed: 2288 (IQR 1480), One Agreed: 2670 (IQR 1300), Neither Agreed: 2498 (IQR 1222). (B) Italian. Median token lengths and IQRs are as follows: Both Agreed: 1068 (IQR 658), One Agreed: 669 (IQR 615), Neither Agreed: 1284 (IQR 336). (C) Spanish. Median token lengths and IQRs are as follows: Both Agreed: 928 (IQR 456), One Agreed: 928 (IQR 99), Neither Agreed: 867 (IQR 144). *P value > 0·05 (Logistic regression).

Supplementary Figure 3. Influence of Note Type on Physician Agreement with GPT-4's Responses.

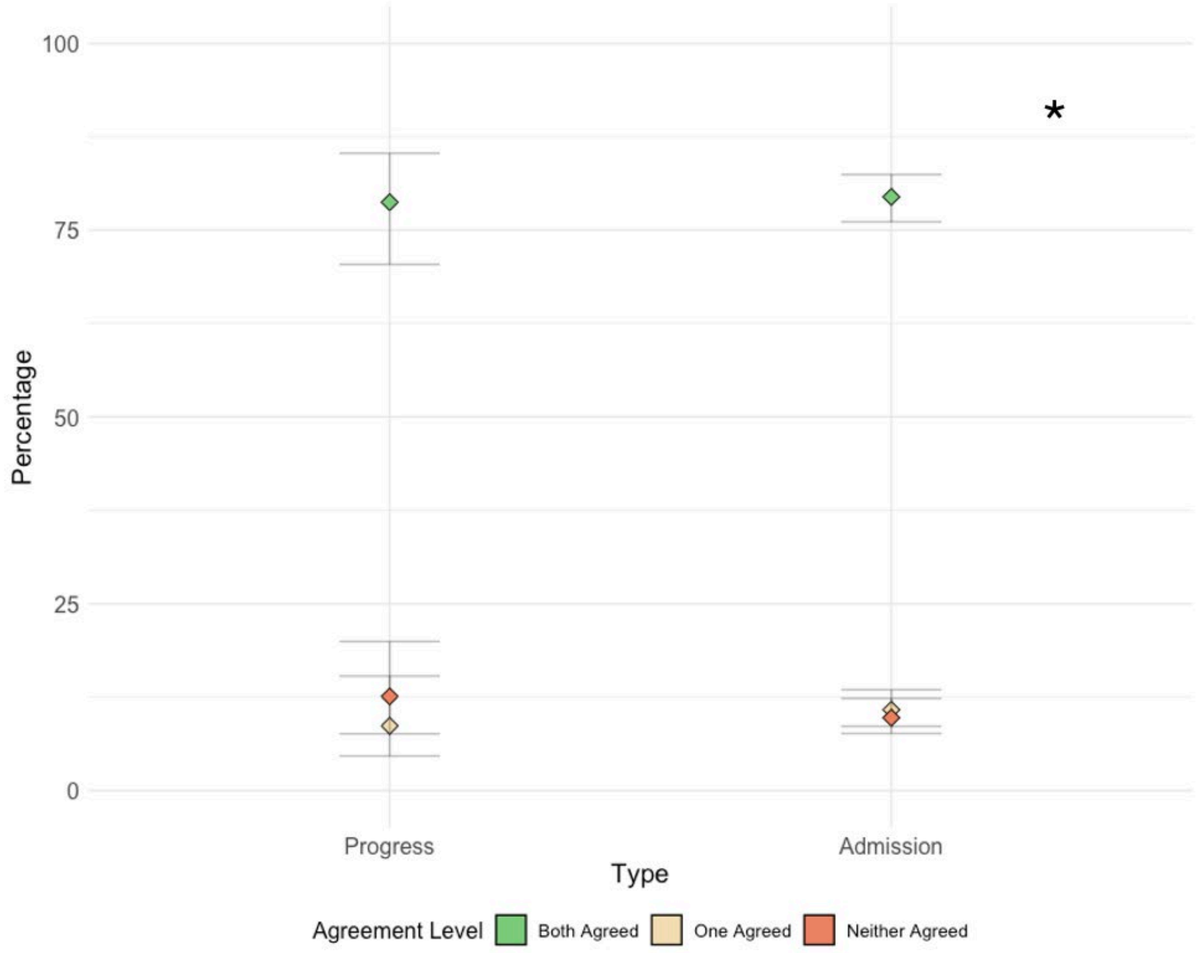

Legend: In progress notes: both physicians agreed with GPT-4's response 79% of the time (100/126, 95%CI 70-85%); only one physician agreed 9% of the time (11/126, 95%CI 5-15%); and neither agreed 12% of the time (15/126, 95%CI 7-19%). In admission notes: both physicians agreed 79% of the time (522/658, 95%CI 76-82%); only one physician agreed 11% of the time (71/658, 95% CI: 9-13%); and neither agreed 10% of the time (65/658, 95%CI 8-12%). Since there was only one consult note, it was treated as a progress note for this analysis. *P value > 0·05 (Chi-squared test).

Supplementary Figure 4. Influence of Country on Physician Agreement with GPT-4's Responses.

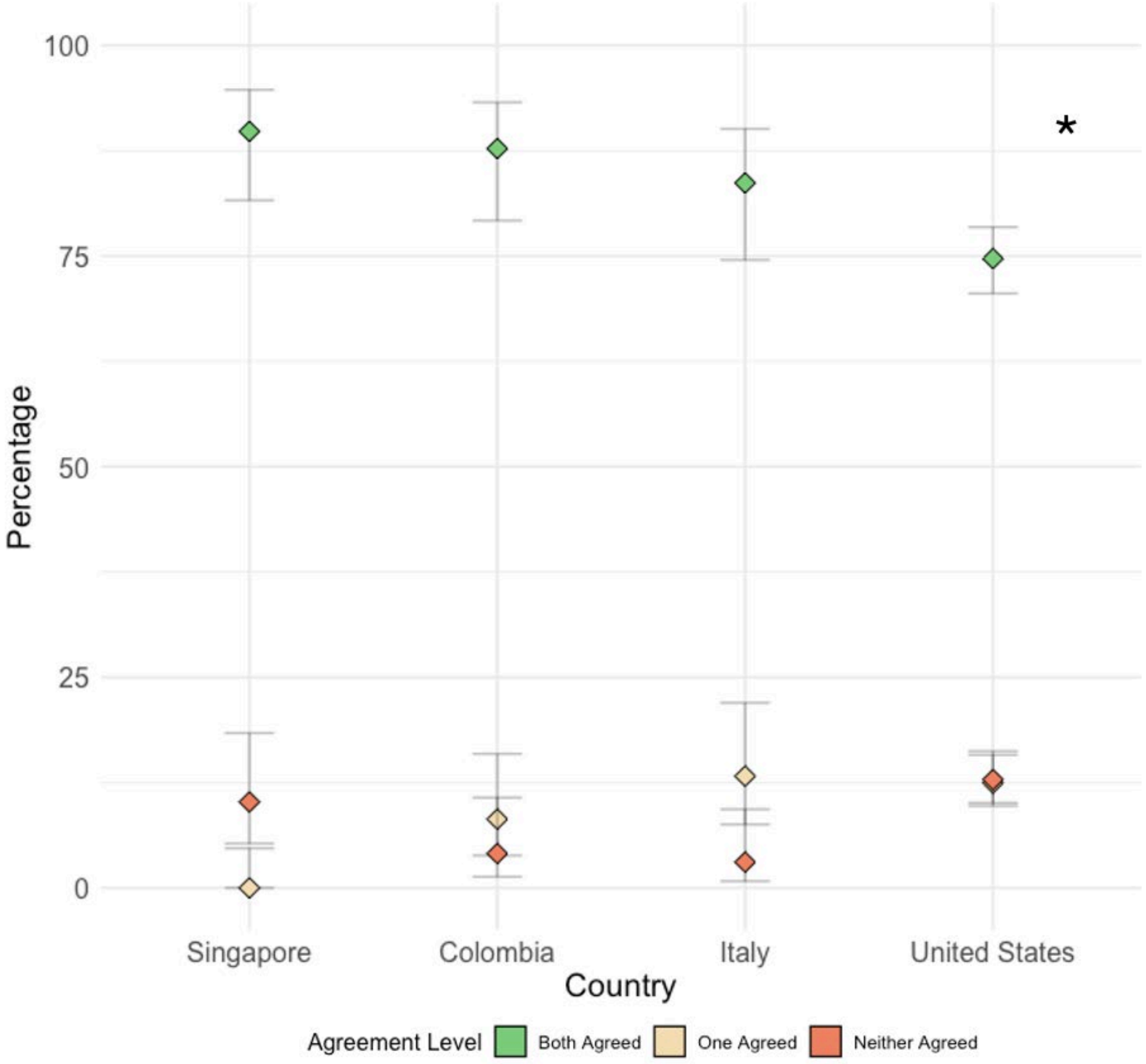

Legend: In Singapore: both physicians agreed with GPT-4's response 90% of the time (88/98, 95%CI 82-95%); and neither agreed 10% of the time (10/98, 95%CI 5-18%). In Colombia: both physicians agreed with GPT-4's response 88% of the time (86/98, 95% 79-93%); only one physician agreed 8% of the time (8/98, 95%CI 4-16%); and neither agreed 4% of the time (4/98, 95%CI 1-11%). In Italy: both physicians agreed with GPT-4's response 84% of the time (82/98, 95% 75-90%); only one physician agreed 13% of the time (13/98, 95%CI 8-22%); and neither agreed 3% of the time (3/98, 95%CI 1-9%). In the United States: both physicians agreed with GPT-4's response 75% of the time (366/490, 95% 71-78%); only one physician agreed 12% of the time (61/490, 95%CI 10-16%); and neither agreed 13% of the time (63/490, 95%CI 10-16%). *P value < 0·001 (Chi-squared test).

Supplementary Figure 5. Analysis of Discrepancies: When Only One Physician Agreed with GPT-4 by Language, Site, and Question.

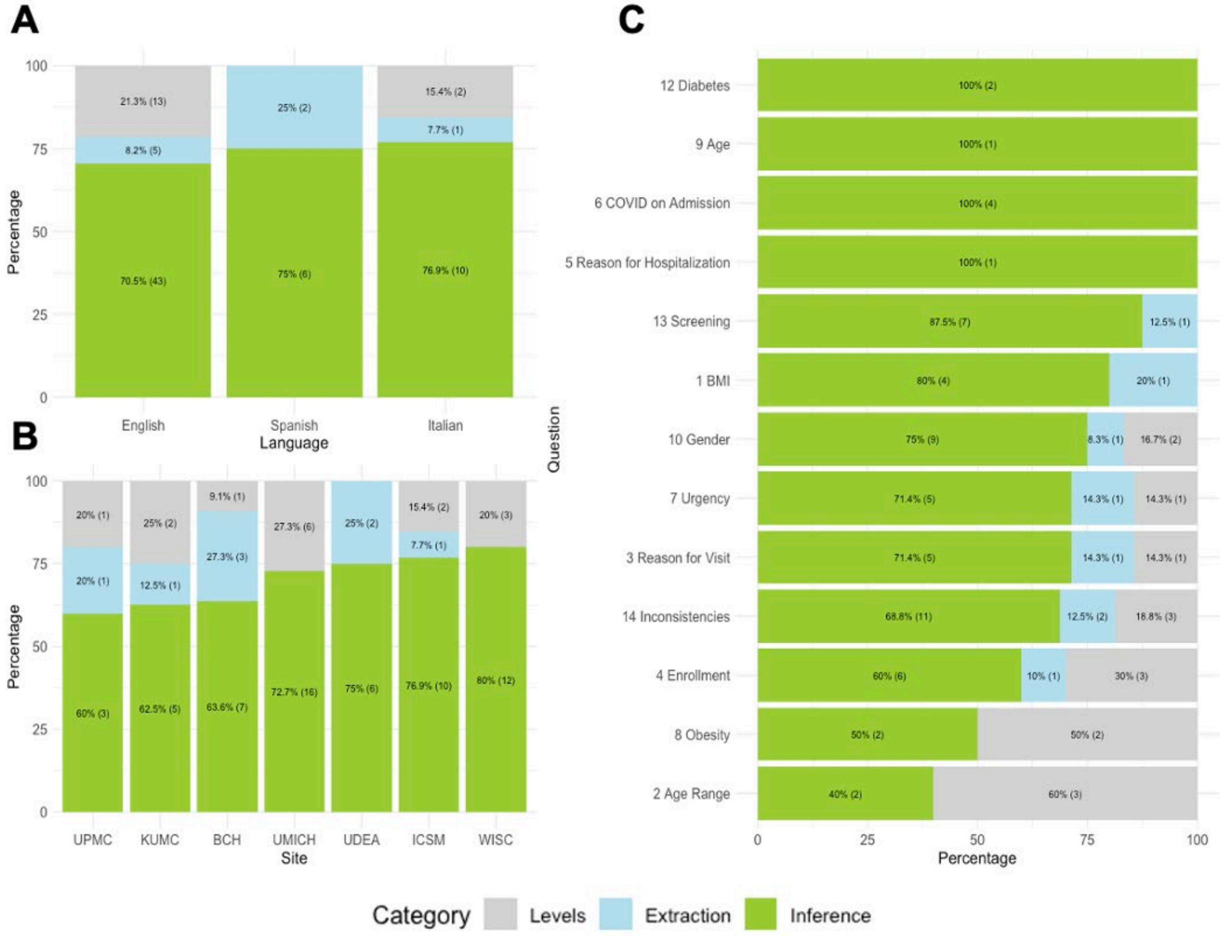

Legend: Categorization of responses when one physician agreed with GPT-4, but the other did not, by Language (A), Site (B), and Question (C). Levels = When both physicians provided the same free-text answer to the question but differed in their agreement with GPT-4's response for unclear reasons. Extraction = When the disagreement between physicians regarding GPT-4's response occurred because one physician overlooked explicit information in the medical note that the other physician noted. Inference = When the difference in physicians' perception of GPT-4's response was due to one physician making different inferences from the medical note than the other physician (e.g., different opinions regarding the need for urgent medical care from symptoms).

Supplementary Figure 6. Analysis of Discrepancies: When Neither Physician Agreed with GPT-4 by Language, Site, and Question.

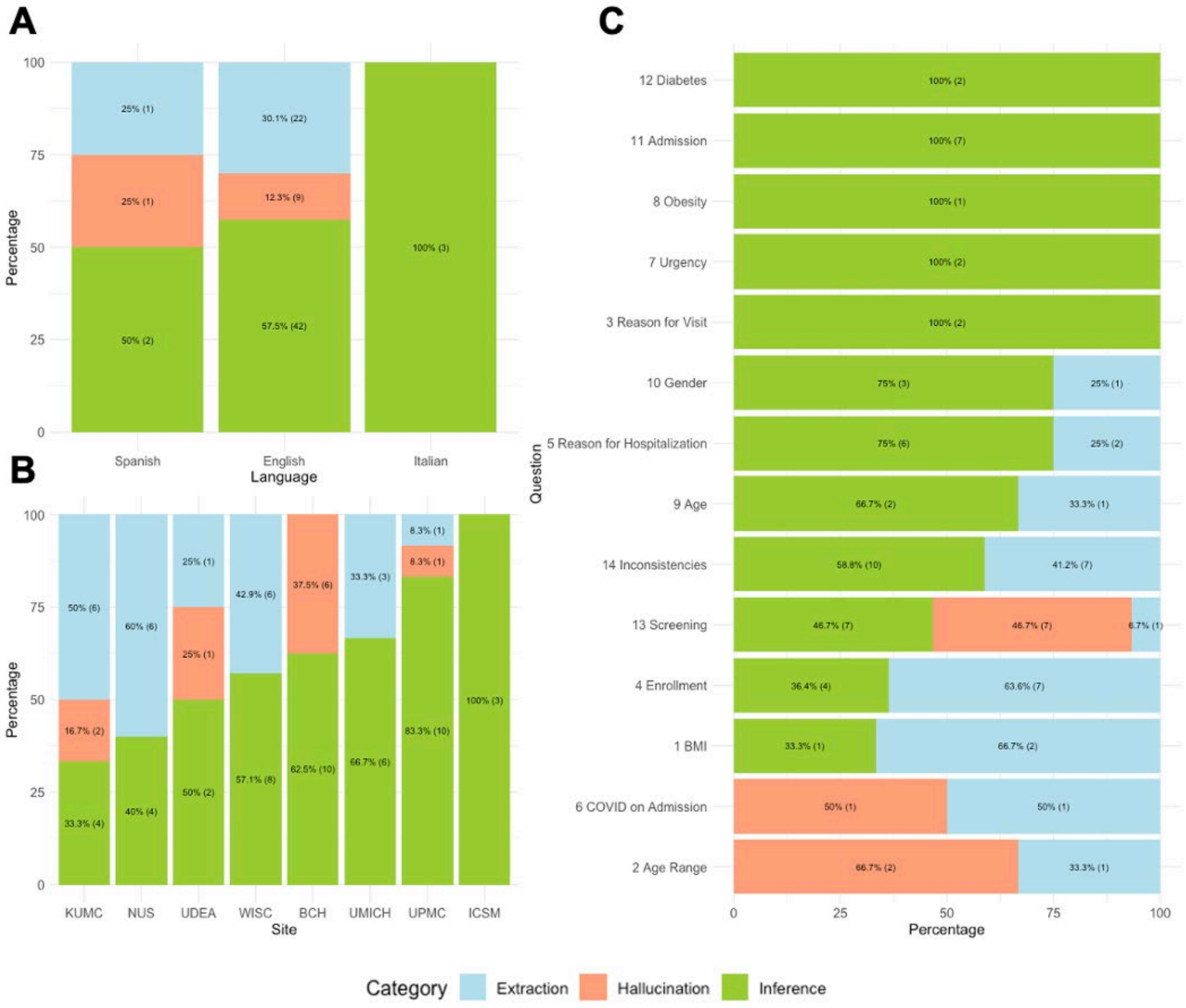

Legend: Categorization of responses when neither physician agreed with GPT-4, by Language (A), Site (B), and Question (C). Extraction = When GPT-4 overlooked explicit information present in the medical note that was noted by both physicians. Hallucination = When GPT-4 generated information that was neither explicitly present nor could be reasonably inferred from the note. Inference = When GPT-4 made or failed to make logical inferences that differed from those made by both physicians.

Supplementary Table 7. Members of the 4CE Consortium.

First Name	Middle Initial	Last Name	Degree(s)	Country
Aaron	J	Masino	PhD	United States
Adeline		Makoudjou	MD	Germany

Adem		Albayrak		United States
Alberto		Zambelli		Italy
Aldo		Carmona		United States
Alexandre		Gramfort		France
Alon		Geva	MD, MPH	United States
Alvar		Blanco-Martínez	MS	Spain
Ana	I	Terriza-Torres	MS	Spain
Anastasia		Spiridou	PhD	United Kingdom
Andrea		Prunotto	PhD	Germany
Andrew	M	South	MD, MS	United States
Andrew	K	Vallejos		United States
Andrew		Atz	MD	United States
Anita		Burgun		France
Anna		Alloni	PhD	Italy
Anna Maria		Cattelan	MD	Italy
Anne Sophie		Jannot		France
Antoine		Neuraz	MD, PhD	France
Antonio		Bellasi	MD, PhD	Switzerland
Anupama		Maram	MS	United States
Arianna		Dagliati	MS, PhD	Italy
Arnaud		Sandrin		France
Arnaud		Serret-Larmande	MD	France
Arthur		Mensch		France
Ashley	C	Pfaff	MD	United States

**Ashley**		**Batugo**	**BS**	**United States**
**Ashok**	**K**	**Krishnamurthy**	**PhD**	**United States**
**Atif**		**Adam**	**PhD, MD, MPH**	**United States**
**Audrey**		**Dionne**	**MD**	**United States**
**Batsal**		**Devkota**		**United States**
**Bertrand**		**Moal**	**MD, PhD**	**France**
**Bing**		**He**	**PhD**	**United States**
**Brendin**	**R**	**Beaulieu-Jones**	**MD, MBA**	**United States**
**Brett**	**K**	**Beaulieu-Jones**	**PhD**	**United States**
**Brian**	**D**	**Ostasiewski**	**BS**	**United States**
**Bruce**	**J**	**Aronow**	**PhD**	**United States**
**Bryce**	**W.Q.**	**Tan**	**MBBS**	**Singapore**
**Byorn**	**W.L.**	**Tan**	**MBBS**	**Singapore**
**Carlo**		**Torti**	**PhD**	**Italy**
**Carlos Tadeu**	**Breda**	**Neto**		**Brazil**
**Charles**		**Sonday**		**United States**
**Charlotte**		**Caucheteux**		**France**
**Chengsheng**		**Mao**	**PhD**	**United States**
**Chiara**		**Zucco**	**PhD**	**Italy**
**Chris**	**J**	**Kennedy**	**PhD**	**United States**
**Christel**		**Daniel**		**France**
**Christian**		**Haverkamp**	**MD**	**Germany**
**Chuan**		**Hong**	**PhD**	**United States**
**Cinta**		**Moraleda**	**MD,PhD**	**Spain**

Clara-Lea		Bonzel	MSc	United States
Damien		Leprovost		France
Daniel	A	Key	BEng	United Kingdom
Daniela		Zöller	PhD	Germany
Danielle		Pillion	MS	United States
Danielle	L	Mowery	PhD	United States
Danilo	F	Amendola	MSc	Brazil
Darren	W	Henderson	BS	United States
Deanne	M	Taylor	PhD	United States
Demian		Wassermann		France
Derek	Y	Hazard	MSc	Germany
Detlef		Kraska	Dr.	Germany
Domenick		Silvio		United States
Douglas	S	Bell		United States
Douglas	A	Murad		United States
Eli	L	Schwamm	BA	
Elisa		Salamanca		France
Emily		Bucholz	MD, PhD, MPH	United States
Emily	J	Getzen	MS	United States
Emily	R	Pfaff	PhD	United States
Emily	R	Schriver	MS	United States
Emma	MS	Toh		Singapore
Enrico	M	Trecarichi	PhD	Italy
Fatima		Ashraf	MS	United States

**Fernando**	**J**	**Sanz Vidorreta**		**United States**
**Francesca**		**Sperotto**	**MD, PhD**	**United States**
**François**		**Angoulvant**	**MD,PhD**	**France**
**Gabriel**	**A**	**Brat**	**MD**	**United States**
**Gael**		**Varoquaux**		**France**
**Giuseppe**		**Agapito**	**PhD**	**Italy**
**Giuseppe**		**Albi**	**MS**	**Italy**
**Griffin**	**M**	**Weber**	**MD, PhD**	**United States**
**Guillaume**		**Verdy**	**MSc**	**France**
**Guillaume**		**Lemaitre**		**France**
**Gustavo**		**Roig-Domínguez**	**MS**	**Spain**
**Hans**	**U**	**Prokosch**		**Germany**
**Harrison**	**G**	**Zhang**	**BA**	**United States**
**Hossein**		**Estiri**	**PhD**	**United States**
**Ian**	**D**	**Krantz**		**United States**
**Jacqueline**	**P**	**Honerlaw**	**RN, MPH**	**United States**
**Jaime**		**Cruz-Rojo**	**MD**	**Spain**
**James**	**B**	**Norman**		**United States**
**James**		**Balshi**		**United States**
**James**	**J**	**Cimino**	**MD**	**United States**
**James**	**R**	**Aaron**	**MHA**	**United States**
**Janaina**	**C.C**	**Santos**	**MS**	**Brazil**
**Jane**		**Newburger**	**MD, MPH**	**United States**
**Janet**	**J**	**Zahner**	**BS**	**United States**

**Jason**	**H**	**Moore**	**PhD**	**United States**
**Jayson**	**S**	**Marwaha**	**MD**	**United States**
**Jean**	**B**	**Craig**	**PhD**	**United States**
**Jeffrey**	**G**	**Klann**	**MEng, PhD**	**United States**
**Jeffrey**	**S**	**Morris**		**United States**
**Jihad**		**Obeid**	**MD, FAMIA**	**United States**
**Jill-Jênn**		**Vie**		**France**
**Jin**		**Chen**	**PhD**	**United States**
**Jiyeon**		**Son**	**MD**	**United States**
**Joany**	**M**	**Zachariasse**	**MD, PhD**	**United States**
**John**		**Booth**	**MSc**	**United Kingdom**
**John**	**H**	**Holmes**	**MS, PhD**	**United States**
**José Luis**		**Bernal-Sobrino**	**MS**	**Spain**
**Juan Luis**		**Cruz-Bermúdez**	**PhD**	**Spain**
**Judith**		**Leblanc**		**France**
**Juergen**		**Schuettler**		**Germany**
**Julien**		**Dubiel**		**France**
**Julien**		**Champ**		**France**
**Karen**	**L**	**Olson**	**PhD**	**United States**
**Karyn**	**L**	**Moshal**	**MBChB, FRCPCH**	**United Kingdom**
**Kate**	**F**	**Kernan**	**MD**	**United States**
**Katie**		**Kirchoff**	**MSHI**	**United States**
**Kavishwar**	**B**	**Wagholikar**	**MBBS, PhD**	**United States**
**Kee Yuan**		**Ngiam**	**MBBS, FRCS**	**Singapore**

**Kelly**		**Cho**	**PhD, MPH**	**United States**
**Kenneth**	**M**	**Huling**	**HS**	**United States**
**Kernan**	**F**	**Kate**	**MD**	**United States**
**Krista**	**Y**	**Chen**	**BS**	**United States**
**Kristine**	**E**	**Lynch**	**PhD**	**United States**
**L. Nelson**		**Sanchez-Pinto**	**MD, MBI**	**United States**
**Lana**	**X**	**Garmire**	**PhD**	**United States**
**Larry**		**Han**	**PhD**	**United States**
**Lemuel**	**R**	**Waitman**		**United States**
**Leslie**		**Lenert**	**MD, MS**	**United States**
**Li**	**L.L.J**	**Anthony**		**Singapore**
**Loic**		**Esteve**		**France**
**Lorenzo**		**Chiudinelli**	**PhD**	**Italy**
**Luca**		**Chiovato**	**MD, PhD**	**Italy**
**Luigia**		**Scudeller**	**MD, MSc**	**Italy**
**Marcelo**	**R**	**Martins**	**MSc**	**Brazil**
**Marcos**	**F**	**Minicucci**	**MD, PhD**	**Brazil**
**Margaret**	**E**	**Vella**	**MPH**	**United States**
**Maria**		**Mazzitelli**	**MD, PhD**	**Italy**
**Maria**		**Savino**	**MS**	**Italy**
**Marianna**		**Milano**	**PhD**	**Italy**
**Marina**	**P**	**Okoshi**	**PhD**	**Brazil**
**Mario**		**Cannataro**	**M.Sc.**	**Italy**
**Mario**		**Alessiani**	**MD, FACS**	**Italy**

**Mark**	**S**	**Keller**		**United States**
**Martin**		**Hilka**		**France**
**Martin**		**Wolkewitz**	**PhD**	**Germany**
**Martin**		**Boeker**		**Germany**
**Maryna**		**Raskin**		**United States**
**Mauro**		**Bucalo**	**MS**	**Italy**
**Meghan**	**R**	**Hutch**	**BS**	**United States**
**Mélodie**		**Bernaux**		**France**
**Michele**		**Beraghi**	**MS**	**Italy**
**Michele**		**Vitacca**	**MD, PhD**	**Italy**
**Miguel**		**Pedrera-Jiménez**	**MS**	**Spain**
**Mohamad**		**Daniar**	**MSIS**	**United States**
**Mohsin**	**A**	**Shah**	**MSc**	**United Kingdom**
**Molei**		**Liu**	**PhD**	**United States**
**Monika**		**Maripuri**	**MBBS, MPH**	**United States**
**Mundeep**	**K**	**Kainth**	**DO, MPH**	**United States**
**Nadir**		**Yehya**	**MD, MSCE**	**United States**
**Nandhini**		**Santhanam**	**MSc**	**Germany**
**Nathan**	**P**	**Palmer**	**PhD**	**United States**
**Ne Hooi Will**		**Loh**	**MBBS**	**Singapore**
**Neil**	**J**	**Sebire**	**MD, FRCPath**	**United Kingdom**
**Nekane**		**Romero-Garcia**	**MD**	**Spain**
**Nicholas**	**W**	**Brown**	**MEng**	**United States**
**Nicolas**		**Paris**		**France**

**Nicolas**		**Griffon**		**France**
**Nils**		**Gehlenborg**		**United States**
**Nina**		**Orlova**		**France**
**Noelia**		**García-Barrio**	**MS**	**Spain**
**Olivier**		**Grisel**		**France**
**Pablo**		**Rojo**	**MD,PhD**	**Spain**
**Pablo**		**Serrano-Balazote**	**MD,MS**	**Spain**
**Paolo**		**Sacchi**	**MD**	**Italy**
**Patric**		**Tippmann**	**MSc**	**Germany**
**Patricia**		**Martel**		**France**
**Patricia**		**Serre**		**France**
**Paula**	**S**	**Azevedo**	**MD, PhD**	**Brazil**
**Paula**		**Rubio-Mayo**	**MS**	**Spain**
**Petra**		**Schubert**	**MPH**	**United States**
**Pietro**	**H**	**Guzzi**	**PhD**	**Italy**
**Piotr**		**Sliz**		**United States**
**Priyam**		**Das**	**PhD**	**United States**
**Qi**		**Long**	**PhD**	**United States**
**Rachel**	**B**	**Ramoni**		**United States**
**Rachel**	**SJ**	**Goh**		**Singapore**
**Rafael**		**Badenes**	**MD, PhD**	**Spain**
**Raffaele**		**Bruno**	**MD**	**Italy**
**Ramakanth**		**Kavuluru**	**PhD**	**United States**
**Richard**	**W**	**Issitt**	**DClinP**	**United Kingdom**

**Robert**	**W**	**Follett**	**BS**	**United States**
**Robert**	**L**	**Bradford**		**United States**
**Robson**	**A**	**Prudente**	**PhD**	**Brazil**
**Romain**		**Bey**		**France**
**Romain**		**Griffier**		**France**
**Rui**		**Duan**	**PhD**	**United States**
**Sadiqa**		**Mahmood**		**United States**
**Sajad**		**Mousavi**	**PhD**	**United States**
**Sara**		**Lozano-Zahonero**	**PhD**	**Germany**
**Sara**		**Pizzimenti**	**MS**	**Italy**
**Sarah**	**E**	**Maidlow**	**AA**	**United States**
**Scott**		**Wong**		**Singapore**
**Scott**	**L**	**DuVall**	**PhD**	**United States**
**Sébastien**		**Cossin**		**France**
**Sehi**		**L'Yi**	**PhD**	**United States**
**Shawn**	**N**	**Murphy**	**MD, PhD**	**United States**
**Shirley**		**Fan**		**United States**
**Siegbert**		**Rieg**	**MD**	**Germany**
**Silvano**		**Bosari**	**Prof.**	**Italy**
**Simran**		**Makwana**	**MS**	**United States**
**Stéphane**		**Bréant**		**France**
**Surbhi**		**Bhatnagar**	**PhD**	**United States**
**Suzana**	**E**	**Tanni**	**PhD**	**Brazil**
**Sylvie**		**Cormont**		**France**

**Taha**		**Mohseni Ahooyi**	**PhD**	**United States**
**Tanu**		**Priya**	**BS**	**United States**
**Thomas**	**P**	**Naughton**	**BA**	**United States**
**Thomas**		**Ganslandt**	**MD**	**Germany**
**Tiago**	**K**	**Colicchio**	**PhD, MBA**	**United States**
**Tianxi**		**Cai**	**ScD**	**United States**
**Tobias**		**Gradinger**	**MD, BSc**	**Germany**
**Tomás**		**González González**	**MD**	**Spain**
**Valentina**		**Zuccaro**	**MD**	**Italy**
**Vianney**		**Jouhet**	**MD,PhD**	**France**
**Víctor**		**Quirós-González**	**MS**	**Spain**
**Vidul**		**Ayakulangara Panickan**	**MS**	**United States**
**Vincent**		**Benoit**	**PhD**	**France**
**Wanjiku**	**FM**	**Njoroge**	**MD**	**United States**
**William**	**A**	**Bryant**	**PhD**	**United Kingdom**
**William**		**Yuan**	**PhD**	**United States**
**Xin**		**Xiong**	**MS**	**United States**
**Xuan**		**Wang**	**PhD**	**United States**
**Ye**		**Ye**	**BMED, MSPH, PhD**	**United States**
**Yuan**		**Luo**	**PhD**	**United States**
**Yuk-Lam**		**Ho**	**MPH**	**United States**
**Zachary**	**H.**	**Strasser**	**MD**	**United States**
**Zahra**		**Shakeri Hossein Abad**	**PhD**	**Canada**
